# Himic Anhydride: A Retro Diels–Alder Reaction for the Organic Laboratory and an Accompanying NMR Study

**DOI:** 10.1021/acs.jchemed.1c00661

**Published:** 2021-11-24

**Authors:** Lee T. Birchall, Sara Shehata, Christopher J. Serpell, Ewan R. Clark, Stefano C. G. Biagini

**Affiliations:** Supramolecular Interfacial Synthetic Chemistry Group, School of Physical Sciences, Ingram Building, University of Kent, Canterbury CT2 7NH, United Kingdom

**Keywords:** Synthesis, Equilibrium, Reactions, Stereochemistry, NMR Spectroscopy, Hands-On Learning/Manipulatives, Organic Chemistry, Laboratory Instruction, Second-Year Undergraduate, Upper-Division Undergraduate

## Abstract

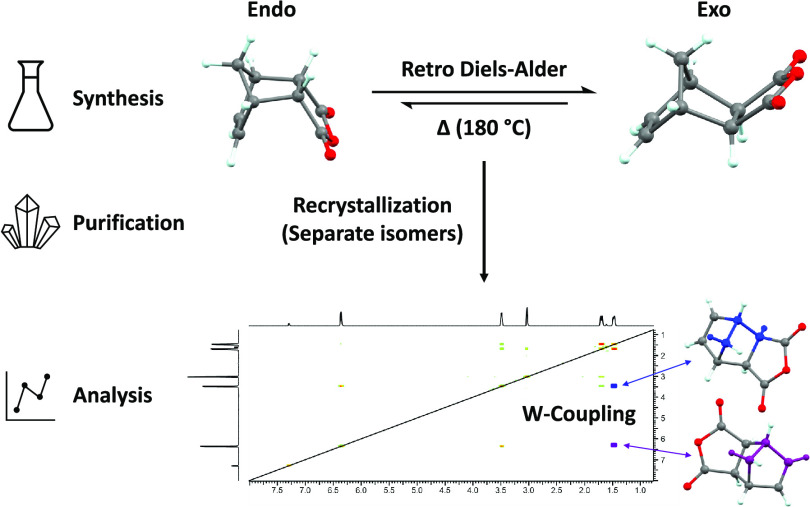

The thermal equilibration of himic anhydride [IUPAC (2-*endo*,3-*endo*)-bicyclo[2.2.1]hept-5-ene-2,3-dicarboxylic acid anhydride] to (2-*exo*,3-*exo*)-bicyclo[2.2.1]hept-5-ene-2,3-dicarboxylic acid anhydride and subsequent recrystallization of the *exo*-product can be performed as a standard undergraduate laboratory experiment requiring minimal equipment. The interpretation of the ^1^H NMR spectra for these norbornene carboxylic anhydride molecules promotes an appreciation of constrained ring systems and factors that affect chemical shifts and coupling constants.

The Diels–Alder^1^ reaction between cyclopentadiene^2^ and maleic anhydride is an established undergraduate experiment. It works well in the time scale of a laboratory session, resulting in good yields of the major isomer which we will refer to as *endo*-himic anhydride but is also known as simply himic anhydride or carbic anhydride.^3^ It is an example of the endo rule seen in practice and can be used to complement or underpin other undergraduate experiments.^4−7^ This article takes the concept of the endo rule further and presents a reaction which requires a better understanding of the concept of kinetic versus thermodynamic control^8−10^ and the theoretical reasons behind this observation. The thermal equilibration of *endo*-himic anhydride to *exo*-himic anhydride (Scheme 1) and subsequent recrystallization of the *exo*-product can be performed in a single day and is suitable as a standard undergraduate laboratory experiment.

**Scheme 1:**
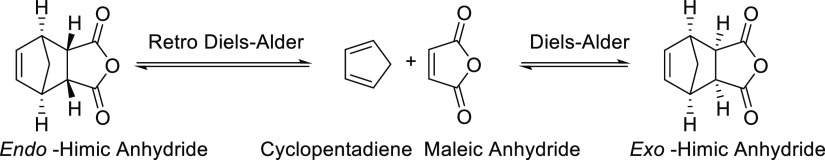
Thermal Equilibration of Himic Anhydride

Because the reaction starts with the Diels–Alder adduct, it avoids cracking of the cyclopentadiene dimer which, although not difficult, requires preparation time and a dedicated fume hood. The interpretation of the ^1^H NMR spectra for these norbornene carboxylic anhydride molecules requires an appreciation of constrained ring systems and factors that affect chemical shifts and coupling constants.^9,11^ In addition, the reaction produces an important starting material for ring opening metathesis polymerization (ROMP) which has become an established approach to synthesize complex macromolecules.^12−19^ Finally, in the context of modules that teach stereochemical concepts, both *endo*- and *exo*-himic anhydrides are each good examples of mesomeric compounds.^20^

Although widely performed, literature preparations regarding the conversion of *endo*- to *exo*-himic anhydride are often either not reported or reported to have relatively modest yields of 16–20%.^21−24^ This reflects some of the challenges that the students will encounter if they pursue research careers and opens discussions about the difference between high yields and useful yields.

## Experimental Overview

An equilibrium mixture of *endo*- and *exo*-himic anhydride can be formed simply by heating *endo*-himic anhydride in the absence of solvent for 1–2 h at 180–200 °C; this typically results in a 55–45% mixture in favor of the *exo*-adduct alongside some minor undetermined impurities. The two isomers can then be separated by repeated fractional crystallization from toluene. Each recrystallization step lowers the overall yield, so there is an inherent challenge in recrystallizing to a high purity in the fewest steps. The relative quantities of each isomer present in sequential recrystallized batches can be determined by ^1^H NMR and/or by GC, and there are options available to allow the experiment to be tailored to different time scales and stages of an undergraduate program.

In both the initial isomerism and each recrystallization step, the students will have ∼90 min reaction time periods. These can be used as an exercise in time management to finish off previous experiments or characterizations, or alternatively, the time can be used to lead students through mechanism and reaction pathways that lead to either the *endo*- or the *exo*-himic anhydride adducts and discuss the thermodynamic implications of each pathway.

## Results and Discussion

This experiment has been trialed with groups of 10–12 Stage 3 UK undergraduate students repeated over a 3 year period. After one recrystallization, sample purity typically ranges between 60 and 80% *exo*-himic anhydride with a yield of 25–50%. After three recrystallizations, purities of 94–98% *exo*-product were found, but, in most cases, a fourth recrystallization was required to achieve a purity of 98% or better. The yields were typically 10–20% (5–10 g of isolated product).

## NMR Interpretation and Workshop

The ^1^H NMR spectra for *endo*- and *exo*-carbic anhydride mixtures give nonoverlapping but similar peaks. Students will have to distinguish which peaks correspond to which isomer via comparison to literature spectra, and this may be used to support training and practice in conducting such searches. Students can then make use of relative signal integration values to estimate the ratios of the two isomers.

The laboratory session can be combined with a workshop session on NMR which includes ^13^C NMR and showcases more advanced NMR techniques such as COSY and HSQC. The ^1^H–^1^H coupling constants about the constrained ring systems are highly dependent upon bond angles and this can be used both to discuss the Karplus relationship and demonstrate the complementarity of NMR and X-ray crystallographic methods.

Further discussion on the interpretation of the spectra follows in the online Supporting Information.

## Learning Outcomes and Assessments

This lab was developed to be the first day of a Stage 3 research project module which required an experiment that was not too challenging to perform and could be carried out without undue time pressure. This was to allow students to familiarize themselves with the research lab setup and equipment and surroundings, and allow supervisors time to address the group collectively and/or individually and show them how to perform procedures that they may have been less familiar with or needed reminding such as GC and NMR sample preparation. If students made an error or the procedure needed to be repeated, there was time to allow for this. It was found that all students were able to perform the reaction and to obtain the product but there were some variations with respect to how many times the students were able to recrystallize the product (typically two recrystallizations were performed).

The recrystallization steps were useful to explain to students the value of purity over quantity and the concept of low but useful yields, particularly in the first step of a synthetic route. The procedure itself is unusual in that standard techniques such as seeding or placing the recrystallizing mixture in an ice-cold bath do not improve the purification process. The students also needed to be mindful of the ratio of solid to solvent to obtain acceptable final yields of the product. Also it was found to be useful to have two methods to compare final purity (GC and NMR). For example many students observed solvent traces in their NMR spectra which were not picked-up on the GC which opened up discussion on levels of analytical purity and precision, and significant figures quoted.

The students obtained ^1^H NMR spectra at the various stages of recrystallization, which resulted in spectra of mixtures. This facilitated guiding students to the various approaches to literature searches of spectra so that they could compare and determine the ratio of the products obtained using NMR integration; this also led to discussions of relaxation times in NMR. The ^1^H NMR spectra do not present peaks explicable with a simple so-called “*n* + 1” rule (actually 2*n*I + 1)^25^ which was useful to explore constrained systems and the Karplus equation.^26^ We found it more practically convenient to supply students with prerecorded examples of the more advanced NMR spectra, rather than run them for each student, to allow them to initially attempt to solve the structures on their own. Following from this lab, a small group session the following week was found to be best to guide the students through the fuller spectroscopic interpretation. The literature searches and NMR interpretation were found to be overwhelmingly useful by students even if some struggled with some of the more advanced concepts as they built a skill set and awareness of resources which were then used repeatedly throughout the subsequent research projects.

## Conclusion

We have developed an undergraduate procedure requiring minimal equipment and resources which trains students in recrystallization techniques and can be linked to taught modules on synthesis and NMR interpretation across a range of undergraduate levels.

## Supporting Information Available

Laboratory notes for students with background information, hazards and safety, and experimental procedure; additional experimental notes for Instructors with chemicals, CAS numbers, equipment needs, synthetic notes, and useful references; NMR and GC interpretation with *endo*- and *exo*-carbic anhydride and mixtures, NMR spectra and interpretations, and GC analyses (PDF, DOCX)
