# Supplementary material for: Himic Anhydride: A Retro Diels–Alder Reaction for the Organic Laboratory and an Accompanying NMR Study
Source: J Chem Educ. 2021 Nov 24;98(12):4013–6. doi: 10.1021/acs.jchemed.1c00661 (PMC8675133; doi:10.1021/acs.jchemed.1c00661)
Supplement: Supplementary file 2 — ed1c00661_si_002.docx [file ed1c00661_si_002.docx]

SUPPLEMENTAL INFORMATION – Lab Documentation For On-line Publication

Himic anhydride: a retro Diels-Alder reaction for the organic laboratory and an accompanying NMR study

Lee T. Birchall, Sara Shehata, Christopher J. Serpell, Ewan R. Clark*, and Stefano C. G. Biagini*.

Supramolecular Interfacial Synthetic Chemistry Group, School of Physical Sciences, Ingram Building, University of Kent, Canterbury CT2 7NH, UK.

E-mail: [s.biagini@kent.ac.uk](mailto:s.biagini@kent.ac.uk); e.r.clark@kent.ac.uk

The information contained within this document is organized in to three sections

**Laboratory notes for students**

Background information, hazards and safety, experimental procedure with references.

**Additional experimental notes for Instructors**

Chemicals, CAS numbers, equipment needs, synthetic notes and references.

**NMR and GC interpretation**

*Endo-* and *exo-*himic anhydride and mixtures. NMR spectral interpretation, GC analysis.

# Laboratory notes for students

The Diels Alder reaction was first described in 1928 and led to the award of the 1950 Nobel prize in Chemistry. The reaction between cyclopentadiene and maleic anhydride is an example of a pericyclic reaction with a concerted mechanism and is classed as a thermally allowed [4 + 2} addition. The major isomer isolated is the *endo* adduct *cis*-5-norbornene-*endo*-2,3-dicarboxylic anhydride, in accordance with the so called endo rule. These reactions are reversible, and the retro Diels-Alder reaction applies the principle of microscopic reversibility to yield appreciable quantities of the corresponding *exo* adduct which is postulated to be thermodynamically similar in energy to the corresponding *endo* isomer. This process was first reported in 1951 and benzene was used as the original solvent for recrystallization.^1^

## Experimental objectives

1. To convert *cis*-5-norbornene-*endo*-2,3-dicarboxylic anhydride to the corresponding *exo* isomer.
2. To purify *cis*-5-norbornene-*exo*-2,3-dicarboxylic anhydride via recrystallization.
3. To analyze and characterize the product via ^1^H NMR, IR, GC, and melting point

## Safety and hazards

- Both *endo* and *exo*-himic anhydride adducts are listed as category 1 sensitizers which may cause allergic reactions. They can cause serious eye irritation and are considered toxic if inhaled or ingested.
- The reaction mixture reaches temperatures of 180-200 ^o^C.
- Toluene is flammable, an irritant, and may cause organ damage on prolonged exposure.
- All weighing, synthetic, and purification procedures must be carried out in a fumehood.
- Appropriate eye protection, laboratory coat, and protective gloves should be worn during this experiment.

# Experimental procedure

| **Compound** | **MW** | **Amount** | **mmol** | **Mp** | **Bp** |
| --- | --- | --- | --- | --- | --- |
| *cis*-5-norbornene-*endo*-2,3-dicarboxylic anhydride | 164.16 | 50 g | 0.30 | 165 °C |  |
| Toluene | 92.14 | 150-250 ml |  |  | 110 °C |

## General Procedure

All procedures to be carried out in a fumehood.

**Thermal Isomerism**

Prepare a heating mantle suitable for heating a 100 ml round bottomed flask and place this above a lab jack on a retort stand.

Weigh *cis*-5-norbornene-*endo*-2,3-dicarboxylic anhydride (50 g) in a 100 ml round bottomed flask. Place the flask in the heating mantle and fit it with a condenser using a Teflon sleeve to avoid contamination with grease. There is no need to run water through the condenser, nor is a magnetic follower required. You should clamp the round bottomed flask at the neck and have the lab jack raised such that the heating mantle can be lowered away from the flask.

Using the heating mantle, gently increase the temperature such that the *cis*-5-norbornene-*endo*-2,3-dicarboxylic begins to melt (~165°C) and until it reaches a temperature of 180 °C. Continue to heat the reaction mixture for 90 minutes, ensuring that the temperature does not drop below 170 °C nor rise above 200 °C. The reaction mixture will turn a golden orange colour due to the formation of thermal degradation products. This degree of colour change is acceptable however if the temperature is allowed to become too high, the reaction mixture will darken considerably and your yield will suffer. The *in situ* temperature can be monitored with infrared/laser thermometers. If these are not available, lower the heating mantle, wait one minute, and then remove the condenser and use a standard laboratory thermometer. Only occasional checking is required.

During the heating process a build-up of white solid on the inside of the glassware may occur. This can be removed by gentle heating of the glass with a heat-gun which will melt the deposits back in to the flask.

Once the heating period is finished, lower the heating mantle away from the flask, remove it, and leave the reaction mixture to cool for 5 minutes (do not shorten this time).

From the top of the condenser add toluene (80 ml) to the reaction mixture (ensure that the heating mantle is not directly below so as to avoid the risk of toluene spilling on to the hot apparatus). Gently swirl the flask to ensure good mixing and allow it to cool to room temperature. NOTE: If a solid has already formed *before* you add the toluene or *immediately* after addition then you will need to add a magnetic follower and heat the mixture until the solid dissolves and then allow the solution to cool to room temperature. Allow the crystals to form slowly without inducement as this will encourage preferential crystallization of the *exo*-isomer.

Once crystallization has occurred, collect the product by vacuum filtration. Wash the crystals with a minimal amount of cold toluene (3-5 ml). Retain a small quantity of sample (50mg) for analytical purposes (^1^H NMR) and recrystallize the crude product from toluene at least twice more using the method below, retaining an analytical sample at each step.

**Recrystallization**

The most efficient way to recrystallize the crude product is to place the product in a round bottomed flask fitted with an air condenser and placed in a heating mantle above a lab jack and add a 1 ml of toluene per gram of sample. Heat the mixture to reflux and, from the top of the condenser, slowly add the minimum amount of further toluene required to dissolve the crystals (usually the same volume again). When adding the additional toluene, you should remove the flask from the heating mantle such as to minimize the risk of toluene spilling on the hot apparatus. NOTE: In some cases, there may be a fine particle residue that does not appear to dissolve and this can be removed by effecting a hot gravity filtration.

For your final sample you should record the melting point. Obtain IR and ^1^H NMR spectra and GC.

## Clean-up

Dispose of any unwanted filtrate in a non-halogenated waste solvent container. Any unwanted product or crude product can be dissolved in wash acetone and disposed in a non-halogenated waste solvent container. Solid residue, solid spillage, and contaminated filter paper can be bagged and placed in a solid waste container. Glassware can be decontaminated with wash acetone or IMS and then washed with soapy water, dried, and returned.

# **Additional experimental notes for Instructors**

## CAS numbers

*cis*-5-norbornene-*endo*-2,3-dicarboxylic anhydride CAS 129-64-6

*cis*-5-norbornene-*exo*-2,3-dicarboxylic anhydride CAS 2746-19-2

toluene CAS 108-88-3

## Commercial supplier:

Carbic anhydride, 99% from Acros Organics [CAS 129-64-6]. On some batches we noticed an impurity is also present in the commercial *endo* carbic anhydride appearing as a fine residue that doesn’t dissolve in toluene. It can be filtered off before the experiment by taking up in toluene, but the subsequent isomerisation is compromised if the toluene is not scrupulously removed so we suggest not to do this but let the students do a hot filtration in due course.

### Other names

Himic anhydride; *cis*-5-norbornene-*endo*-2,3-dicarboxylic; (2-*endo*,3-*endo*)-bicyclo[2.2.1]hept-5-ene-2,3-dicarboxylic acid anhydride

## Sample Write-up

*Endo*-himic anhydride (50 g, 0.305 mol) was added to a round bottomed flask with condenser attached. The solid was heated (170 – 190 °C, 2 h) gently to avoid charring. After heating, the orange liquid was left to cool slightly and then toluene (80 ml) was added. Some of the liquid solidified due to the cold toluene and so the solution was heated to reflux until all solid had dissolved. The solution was left to cool slowly to room temperature leaving the flask alone for crystallization to occur. Once crystallization had occurred, the solvent was carefully decanted off and the product was collected by vacuum filtration, washed with toluene (3 ml), and allowed to dry to give crude product (22 g) as colourless crystals which were analysed by ^1^H NMR.

## Recrystallization

The crude product was dissolved in toluene in a ratio of 1:2 in g:ml and heated until all solid had dissolved. There were still fine particles present after swirling and heating, so a hot filtration was performed. The hot filtrate was left to cool and crystallization occurred. The solvent was decanted off and the product collected by vacuum filtration. The product was washed with cold toluene (3 ml) and allowed to dry before being weighed and analysed by ^1^H NMR.

The recrystallization process was repeated twice more to yield the product as a white crystalline solid (6.5 g, 40 mmol, 13%).

*cis*-5-norbornene-*exo*-2,3-dicarboxylic anhydride literature^1^ melting point 142^o^C.

## Melting points

| % Endo in Exo | T_m_ (°C) 1 | T_m_ (°C) 2 | T_m_ (°C) 3 | T_m_ (°C) 4 |
| --- | --- | --- | --- | --- |
| 100 | 164 – 166 | / | / | / |
| 75 | 136 – 139 | 136 – 141 | 136 – 141 | 132 - 141 |
| 50 | 112 – 114 | 112 – 115 | 112 – 115 | 112 – 115 |
| 25 | 111 – 121 | 112 – 128 | 110 - 130 | 112 – 128 |
| 0 | 143 – 145 | / | / | / |

Table 1 Melting points of mixtures of endo- and exo-cis-5-norbornene-exo-2,3-dicarboxylic anhydride as determined experimentally

## Synthetic notes

The procedure^2^ was used in our labs as part of undergraduate projects where significant quantities of the *exo*-himic anhydride were required for subsequent synthetic steps.^3–5^ The procedure described can be halved in quantities without any issue, *i.e.* starting from 25 g of the *endo*-himic anhydride and we would recommend this for a stand-alone experiment. However when using 10 g or less of starting material, we found that some students struggled with the recrystallization steps, typically using too much toluene and so failing to recover crystals.

90-120 minutes is the optimum time for the isomerisation but this can be shortened to 60 minutes and still result in appreciable yields of the product.

If the temperature is kept below 170 ^°^C, the reaction will not work as the rate of the retro Diels-Alder reaction will be too slow. If the temperature is raised above 200 ^°^C, it will result in a black reaction mixture containing an appreciable amount of insoluble residue and it can be difficult for a student to rescue this. If it happens at an early stage then we suggest starting again.

We advise that the reaction mixture not be allowed to cool to room temperature before the addition of toluene. This is because it solidifies to a hard solid mass that is difficult and time-consuming to dissolve in toluene. The solid mass can be melted by heating but care should be taken not to char the mixture.

We did not find any advantage during recrystallization procedures of cooling below room temperature in an ice bath as this encourages the unwanted *endo*-himic isomer to also crystallize.

We also did not find any advantage in agitating the mixture, scratching or seeding the solutions with pure *exo*-isomer as these processes appear to also encourage the nucleation of the *endo*-isomer and in our hands were ineffective at giving selective recrystallization. The first recrystallization of the reaction mixture is usually the slowest and can take between 30-60 min. The more impure the mixture the longer the recrystallization process which is another reason why charred mixtures are difficult to rescue.

The remaining solvent that is decanted off during each of the steps above can be added together, concentrated *in vacuo* and the isomerisation procedure repeated to improve overall yield if desired. However if this is done, the filtrate should be dried as much as possible otherwise the toluene will have a negative impact of the subsequent isomerization leading to a charred reaction mixture.

The recrystallization steps have also been reported to have been carried out in acetone^6^ rather than toluene but we have not repeated this.

Timescale options

We recommend that the reaction be done as part of an all-day lab (6 hours) and that there be scope for the recrystallization steps to be carried out in a subsequent session. This will reinforce the time management aspect of the chemical laboratory practice. However, the practical can be adapted to a shorter session as follows:

3h Carry out one reaction for 90min and effect one recrystallization . Analyse product by ^1^H NMR.

# **NMR and GC interpretation notes for Instructors**

### ^1^H NMR analysis

The annotated ^1^H NMR and COSY of commercial *endo*-himic anhydride are shown below:

Figure 1 Annotated ^1^H NMR of endo-himic anhydride as bought from Acros Organics, conducted in CDCl_3_ at 298 K and referenced to residual solvent.

Figure 2 Annotated COSY NMR of endo-himic anhydride as bought from Acros Organics, conducted in CDCl_3_ at 298 K.

The easiest peak to assign for himic anhydride is peak 1 as the hydrogens on the alkene are deshielded as a result of the π-bond and it is well documented that hydrogens on an alkene appear within this ppm range. This is therefore a good starting point for piecing the molecule together using COSY NMR. From the COSY, environment 1 couples to peak 2 and also shows weak coupling to peak 3”. From this we can deduce that 2 is the hydrogen on the adjacent carbon as three bond coupling is strong and long-range coupling is not normally observed. However, in this rare case, long-range coupling between the alkene hydrogen and the bridgehead hydrogen, 3”, is observed due to W-coupling. W-coupling is seen in rigid systems like himic anhydride where the four bonds (H – C – C – C – H) are stuck in a ‘W-like’ arrangement where there is weak overlap between the anti-bonding orbitals of both C-H bonds. This phenomenon allows the bridgehead hydrogens to be distinguished, which are in chemically different environments due to the rigidity of the compound.

In non-rigid compounds where free rotation about C-C and C-H bonds is allowed, molecules move rapidly between possible conformations and the chemical shift value observed in the ^1^H NMR is an average value associated with the environments experienced by the protons. For most alkyl fragments, the protons on a carbon move into and out of all the same environments and so give rise to a single signal as they are equivalent over time. In rigid compounds such as himic anhydride where free rotation cannot occur, the hydrogens are in chemically distinct environments and second-order effects are observed. The two protons can never interconvert without breaking the molecule and since their environments are different, so too are their chemical shifts. As the two hydrogens 3’ and 3” are not in chemically equivalent environments, their peaks are separate and they couple with each other, resulting in the two observed doublet peaks, 3’ and 3”. As a result of this coupling and because they are in similar environments and so have similar chemical shifts, they exhibit ‘roofing’ (Figure 3 below) .which is distinctive of systems exhibiting second order effects. The degree of this ‘roofing effect’ depends on the similarity in the chemical environments of the protons. In systems where the protons are nearly isochronous the ‘roofing effect’ is more pronounced. In the case of *endo*-himic anhydride, the roofing is moderate as the two environments are comparatively different.

Figure 3 Representation of the 'Roofing effect' seen in ^1^H NMR spectra as a result of second order effects.

The assignment of peak 3” is confirmed further by the presence of three-bond coupling to peak 2 which can be seen in the COSY spectrum but is too weak to be seen on the 1D ^1^H NMR spectrum. This weak coupling can be understood using the Karplus equation^7^ which describes the relationship between the dihedral angle in a three-bond coupling and the expected magnitude of the coupling. According to the Karplus equation, a dihedral angle of 90° will give the lowest possible coupling constant, *J*. Deviation from 90° towards 180° gives the greatest coupling constants due to the antiperiplanar conformation of the bonds that provides the best overlap of antibonding orbitals. Deviation towards syn-periplanar at 0° also provides greater coupling constants compared to 90°, but the overlap is not as efficient as an antiperiplanar system. Using free-for-academic use CCDC Mercury software^8^ and the crystal structure of *endo-*himic anhydride^9^, the dihedral angle between hydrogens 3” and 2 was calculated to be 65.2°. An estimation^10^ of the corresponding coupling constant yields a value of 2 Hz and in practice the coupling constant, *J*, is so small that it is not observable by ^1^H NMR under the conditions used.

Once peak 3” has been assigned, peak 3’ can subsequently be assigned as it has both the two-bond germinal coupling to peak 3” and the three-bond vicinal coupling to peak 2 which are shown by the COSY. Unlike with hydrogen 3”, the coupling between hydrogen 3’ and 2 is strong enough to be seen by 1D ^1^H NMR, resulting in peak 3” being a doublet of triplets. This is further confirmed by calculations from the crystal structure which shows the dihedral angle between hydrogen 3’ and 2 to be 61.7°. Despite it only getting closer to 0° compared with the dihedral angle between hydrogen 3” and 2 by only 3.5°, it is enough to increase the strength of the *J* coupling, making it visible by ^1^H NMR. However, the *J* coupling is still weak at *J* = 1.58 Hz.

Unlike with hydrogen 3” which exhibits long-range W-coupling to hydrogen 1, hydrogen 3’ does not show the same long-range coupling behavior as there is no suitable hydrogen in an appropriate geometric relationship.

Finally, peak 4 can be assigned as it is coupled only to peak 2 in the COSY which agrees with expectation as hydrogen 2 is the only hydrogen three bonds away to which it could couple. Furthermore, as described above, there is no long-range coupling between hydrogen 4 and 3’. Despite the COSY only showing hydrogen 4 coupling with hydrogen 2, the ^1^H NMR shows a quartet of peaks exhibiting second order effects.

On the other side of the molecule, hydrogen 1 shows an apparent triplet (i.e. an overlapping doublet of doublets with similar *J* values), which we have assumed to be from the coupling with hydrogens 2 and 3” which happen to have similar coupling constant values. The magnitude of the coupling is *J* = 1.83 Hz and the dihedral angle between the coupling protons 1 and 2 is 21.4° as calculated from the crystal structure. As expected, the coupling is greater than that between protons 2 and 3’ which had a dihedral angle of 61.7°. However, even with such a large change in dihedral angle the coupling is not much greater in magnitude. This can be put down to the *syn* relationship not providing good orbital overlap, even at an angle like 21°.

Students presented with a ^1^H NMR spectrum alone of *endo*-himic anhydride would not be expected to be able to differentiate between the peaks corresponding to 3’ and 3” or between the peaks for 2 and 4.

Having assigned the ^1^H NMR of the *endo* isomer, the ^1^H NMR of the *exo* isomer can be examined and compared:

Figure 4 Annotated ^1^H NMR spectrum of exo-himic anhydride, conducted in CDCl_3_ at 298 K and referenced to residual solvent.

Figure 5 Annotated COSY NMR spectrum for exo himic anhydride, conducted in CDCl_3_ at 298 K and referenced to residual solvent.

As with the *endo* isomer, the protons on the alkene are easiest to assign as peak 1 for the same reasons and therefore can be used as a good starting point to assign the ^1^H NMR spectrum using the COSY spectrum. From the COSY, peak 1 can be seen to be coupling to peak 2 strongly and peak 3”weakly. As with the *endo*- isomer, 2 must therefore be the proton environment on the carbon adjacent to 1 due to the three-bond coupling. Furthermore, some very weak W-coupling is present with proton 3”, which distinguishes proton environment 3” from 3’ on the bridgehead. Having assigned peak 3”, peak 3’ can be assigned as the other bridgehead proton, which can further be confirmed by its coupling to peak 4 on the COSY.

This coupling to peak 4 is another example of W-coupling which is stronger than that between 1 and 3”, due to the less distorted ‘W-like’ arrangement. This is different from the *endo* isomer where there was no W-coupling with the bridgehead proton closest to the alkene. This provides strong evidence for the *exo* geometry, as the ‘W-like’ arrangement for W-coupling is only possible in the *exo* isomer. Having deduced the assignment of peak 4 through process of elimination and its W-coupling to peak 3’, it is interesting to note that the COSY shows no sign of 4 coupling to 2 through a common three-bond coupling. This seems strange at first but using the crystal structure of the *exo* which was collected (Figure 6), the dihedral angle between 2 and 4 was found to be 75°. This 75° angle provides very little orbital overlap for coupling to occur, estimated^10^ at 0.7 Hz, therefore explaining why none was seen on the COSY. Furthermore, it makes sense why peak 4 is a doublet, as it is only visibly coupling to peak 3’ through the W-coupling as described, with J = 1.46 Hz. As for the endo isomer, students presented with a ^1^H NMR spectrum alone of *exo*-himic anhydride would not be expected to be able to differentiate between the peaks corresponding to 3’ and 3” or between the peaks for 2 and 4.


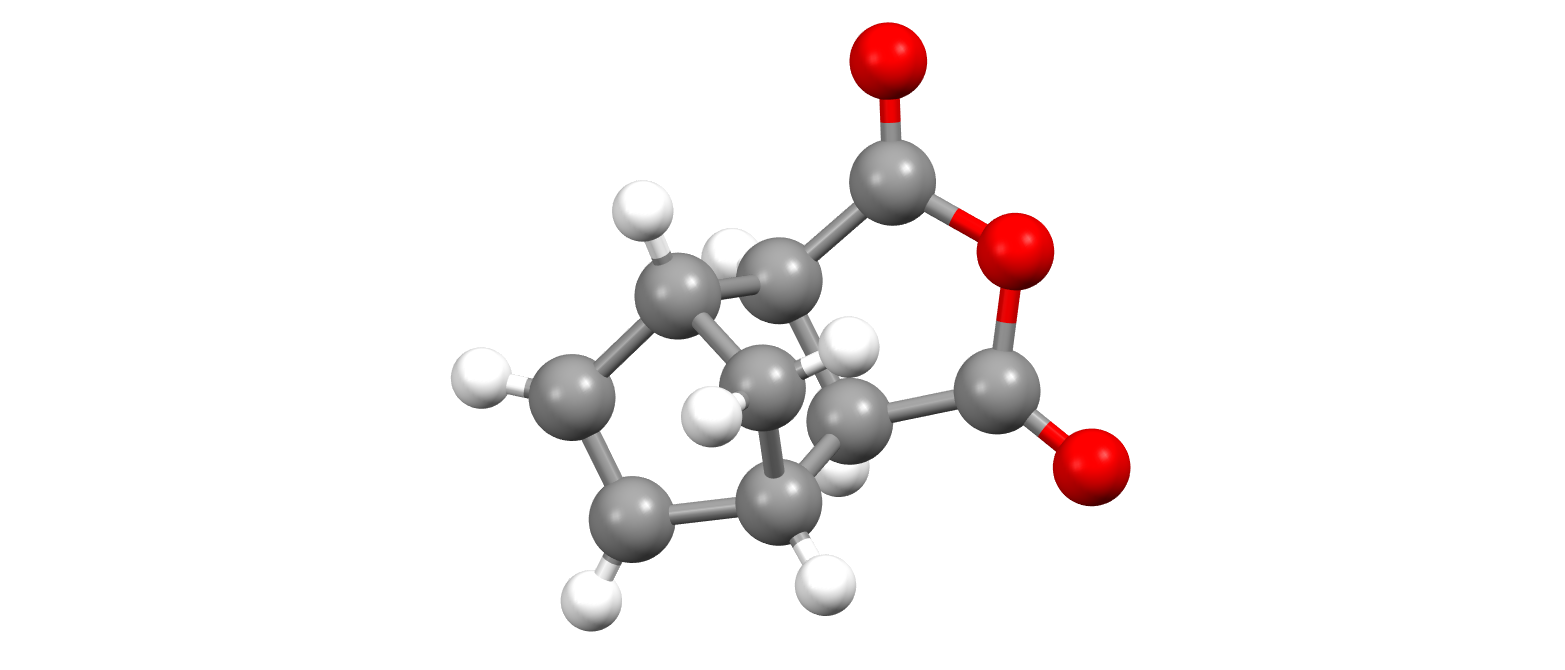


Figure 6 Crystal structure of the exo isomer of himic anhydride.

The remaining peak at δ = 1.61 is assigned to adventitious water.

### GC-MS analysis

Having confirmed the formation of *exo-*himic anhydride by single crystal XRD and ^1^H NMR, GC-MS measurements were run on the same samples, providing the following results:


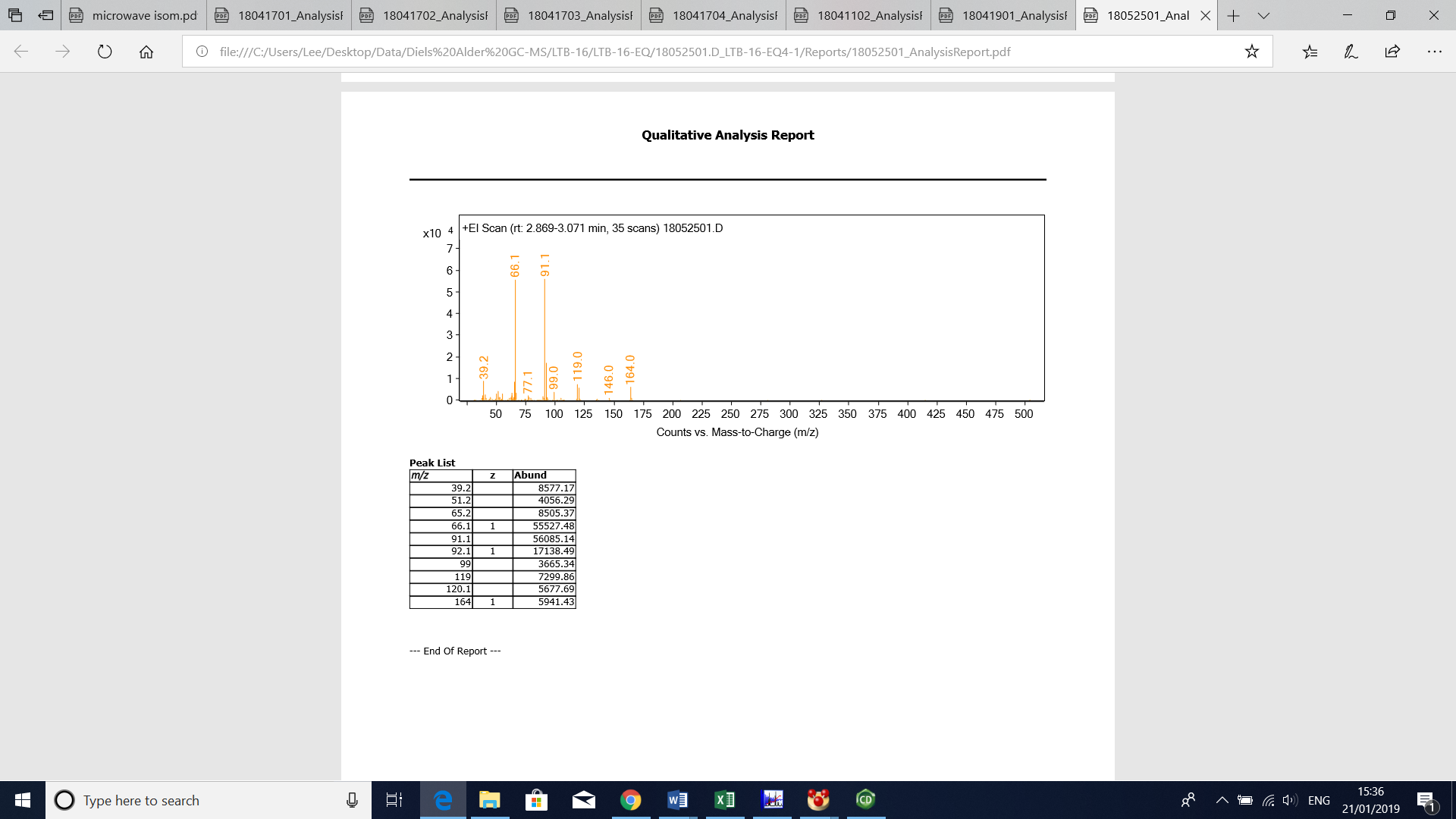

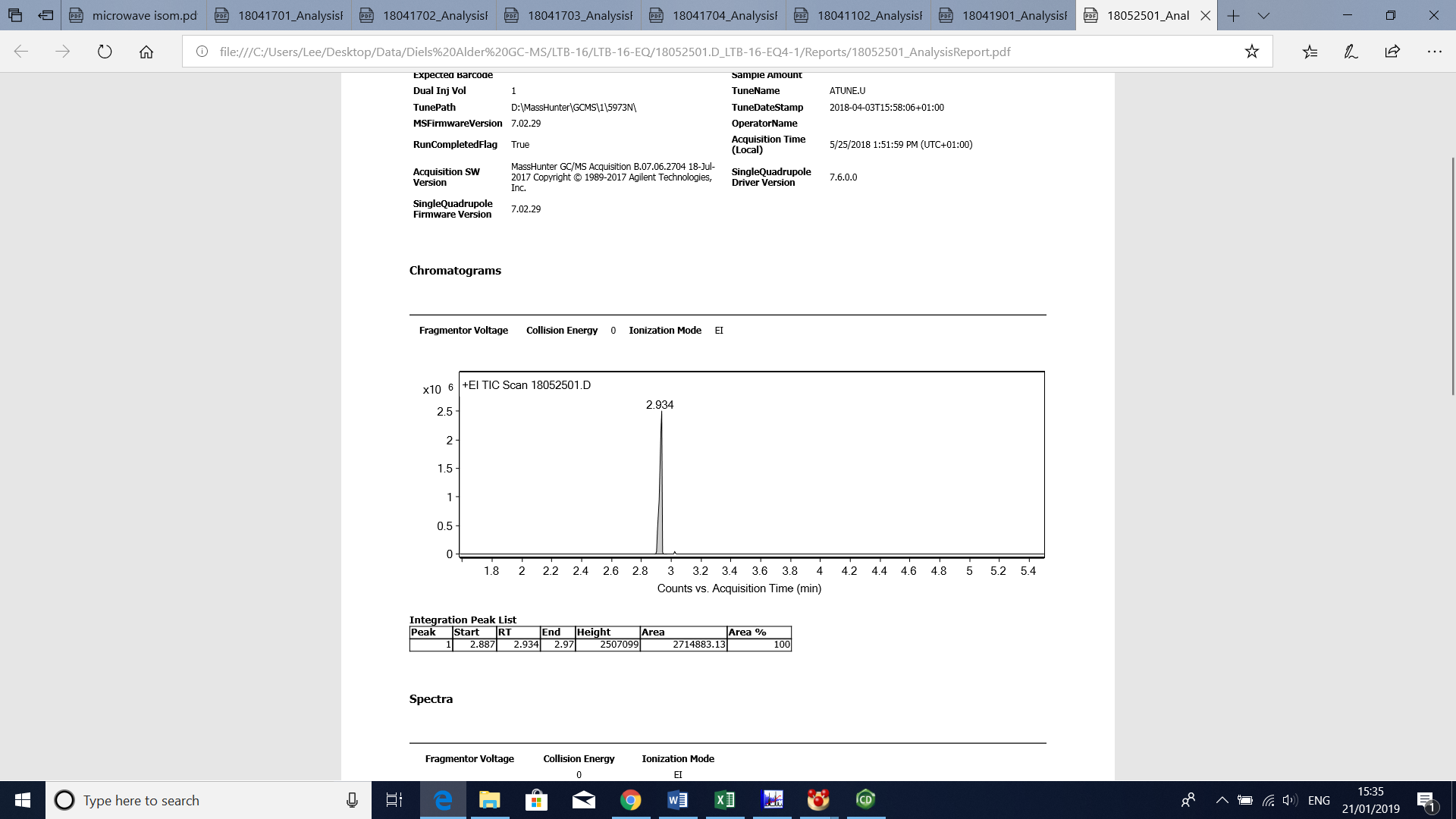


Figure 8 Mass spectrum of purified exo-himic anhydride.

Figure 7 GC trace of purified exo-himic anhydride.

In the GC trace above, the main peak at 2.9 min is the *exo* isomer. A small peak is visible just after 3 min which corresponds to a trace of the *endo* isomer, however it was not considered large enough by the software to be integrated. Therefore, it is reasonable to say that this sample is >99% pure. The mass spectrum of *exo*-himic anhydride (Figure 8) shows the parent ion peak at m/z = 164, the monoisotopic mass of himic anhydride. The peak at m/z = 66.1 is indicative of cyclopentadiene.

Having obtained GC-MS results from a sample of pure *exo*, a crude sample was run on the GC-MS in order to ensure that the *exo* and *endo* isomers had different retention times. The results of the GC-MS analysis of the crude sample along with a ^1^H NMR of the same sample are shown below:


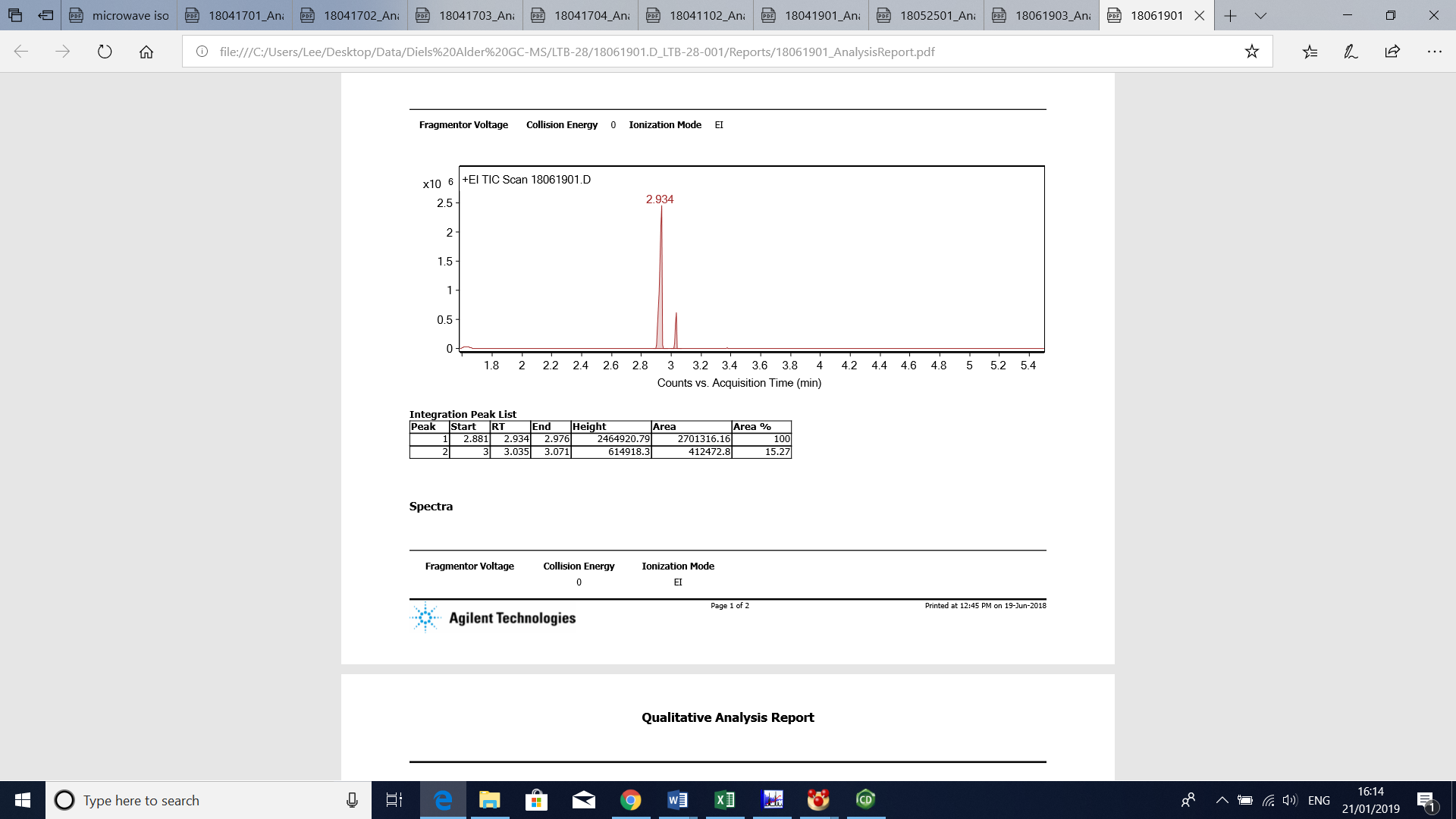


Figure 9 GC trace of a crude sample containing both exo- and endo-himic anhydride.

Figure 10 ^1^H NMR spectrum of a crude sample containing both exo- and endo-himic anhydride, conducted in CDCl_3_ at 298 K and referenced to residual solvent.


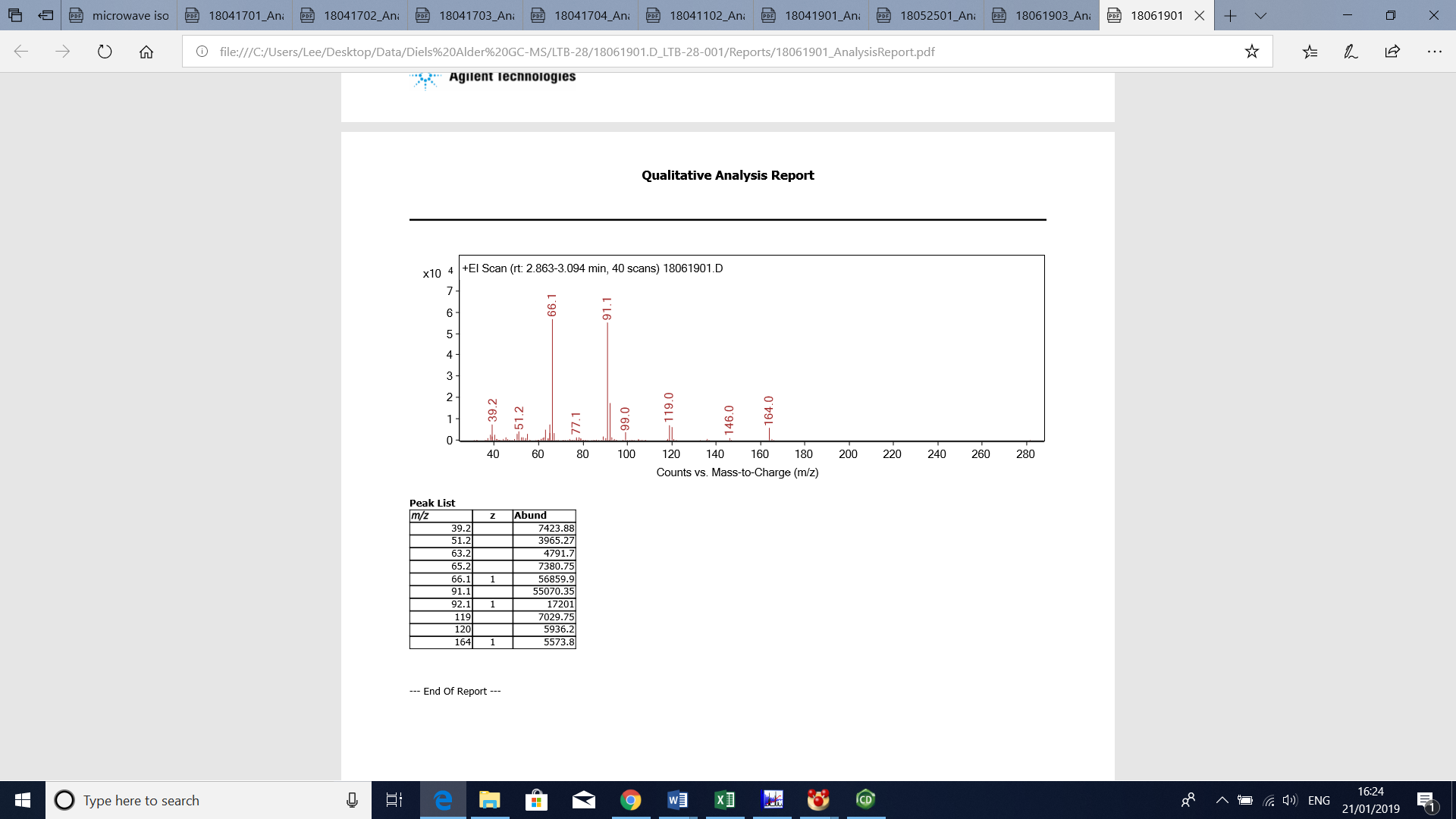


Figure 11 Mass spectrum of the crude sample containing both exo- and endo-himic anhydride.

From the ^1^H NMR of the crude mixture (Figure 10), it is possible to see that there is more *exo* present than *endo*. The most distinctive peak that indicates *exo* is the peak at δ = 3.03 as it is the environment that changes the most when comparing *endo* to *exo*. The alkene peak is in almost the identical place and the bridgehead peaks are shifted slightly upfield in the *exo* compared to the *endo*. By looking at the GC trace (Figure 9), the peak at 3.0 min is much larger in the crude sample, compared to the pure sample as would be expected. The ratios of the peaks in the NMR spectrum are also similar to the ratios found in the GC trace, indicating that the peak at 3.0 min in the GC is in fact the *endo*. This was further confirmed by the MS (Figure 11) which contained the parent ion peak for himic anhydride at m/z = 164.0. It is also worth noting that upon inspection of a crude crystalline mixture containing both *endo* and *exo* under microscope, the isomers can be distinguished by their morphology. A plate-like crystal from the crude was screened by single crystal XRD and gave parameters indicative of the *endo*. A needle-like crystal was then screened by single crystal XRD and gave parameters indicative of the *exo*.

Using the GC-MS integration values calculated by the software for the crude mixture above as an example, the % of *exo* in the sample can be determined as shown below:

$${Exo}_{\%}= \frac{{Area \%}_{RT=2.934(Exo)}}{{Area \%}_{Total}}\times100= \frac{{Area \%}_{RT=2.934(Exo)}}{{Area \%}_{RT=2.934(Exo)}+{Area \%}_{RT=3.035(Endo)}}\times100$$

$$=\frac{100}{100+15.27}\times100=86.8 \%$$

Equation 1 Calculation of purity of exo-himic anhydride using GC analysis.

### T_1_ analysis and GC-MS comparison

Measurement of the spin-lattice relaxation time, T_1_, was performed on a crude mixture of *endo*- and *exo*- species to confirm that the integration values from ^1^H NMR could be used to determine an approximate ratio of *exo*:*endo*. Peaks from the two different molecules that have very similar T_1_ values can be used to determine the compound ratios via comparative integration. Peaks with very dissimilar T_1_ values will not give very accurate ratios and should not be used. Some results from the T_1_ measurements are shown below:


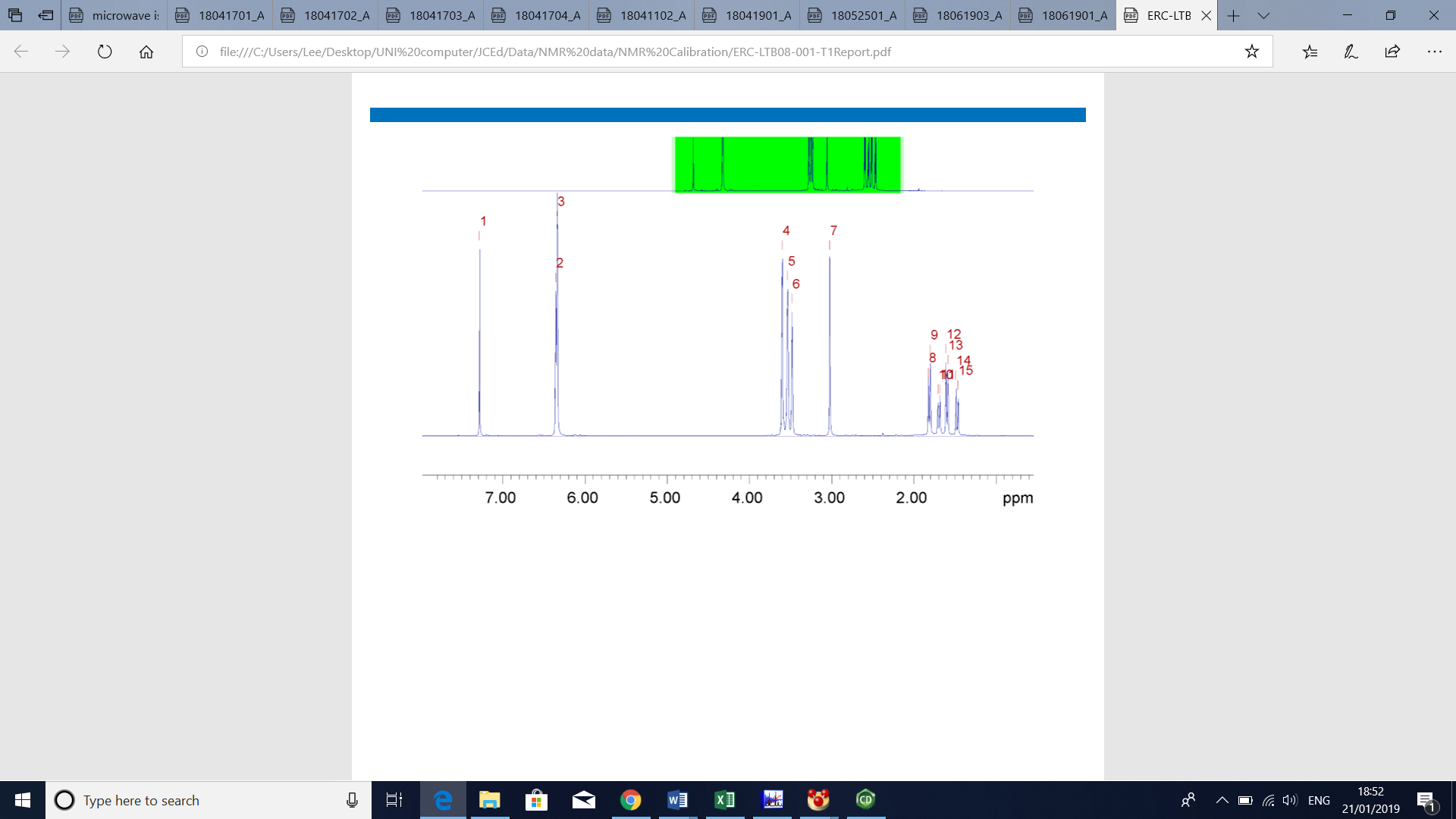


Figure 12 Annotated ^1^H NMR of a crude sample containing both exo- and endo-himic anhydride for T_1_ analysis, conducted in CDCl_3_ at 298 K and referenced to residual solvent.

Table 2 T_1_ values for each individual peak that is labelled in Figure 12 along with their respective error values.

| Peak Name | F2 (ppm) | T1 (s) | error |
| --- | --- | --- | --- |
| 1 residual CHCl_3_ | 7.284 | 7.78 | 0.1368 |
| 2 *endo* or *exo* H_1_ | 6.355 | 5.10 | 0.02613 |
| 3 *endo* or *exo* H_1_ | 6.338 | 5.50 | 0.02732 |
| 4 *exo* H_2_ | 3.600 | 5.01 | 0.02700 |
| 5 *endo* H_4_ | 3.535 | 4.83 | 0.01921 |
| 6 *endo* H_2_ | 3.481 | 4.96 | 0.02585 |
| 7 *exo* H_4_ | 3.021 | 5.40 | 0.02017 |
| 8 *exo* H_3’_ | 1.820 | 2.89 | 0.01266 |
| 9 *exo* H_3’_ | 1.798 | 2.88 | 0.01205 |
| 10 *endo* H_3’_ | 1.705 | 2.57 | 0.007416 |
| 11 *endo* H_3’_ | 1.679 | 2.59 | 0.008450 |
| 12 *exo* H_3”_ | 1.607 | 2.74 | 0.01236 |
| 13 *exo* H_3”_ | 1.586 | 2.74 | 0.01720 |
| 14 *endo* H_3”_ | 1.486 | 2.70 | 0.01505 |
| 14 *endo* H_3”_ | 1.460 | 2.72 | 0.01432 |

From the T_1_ results (Table 2), the peaks that can be compared to give the best approximate ratio are peaks ‘12 & 13’ (2.74 & 2.74) and ‘14 & 15’ (2.70 & 2.72) which are both indicative of the same bridgehead proton environment in the *endo* and *exo* respectively. The difference in T_1_ between 12 and 14 is only 0.04 s and between 13 and 15 only 0.02 s. Therefore, comparing the ^1^H NMR integration values of 13 and 15 from the crude sample measured by GC-MS above, which are 0.09 and 0.44 respectively, results in an *exo* content of 83 %.

The values for the % of *exo*, determined both by GC-MS (86.8 %) and from the NMR integration calculations (83 %) are only different by around 4 %. Therefore, it is possible to use the integration of the peaks mentioned above to quickly calculate an approximate *exo* % for those without easy access to GC-MS.

### Assigning the ^13^C NMR:

The ^13^C NMR spectra for *endo*- and *exo*-himic anhydride were assigned using low-resolution HSQC and DEPT spectra. An example of how these techniques were used is shown and described below in Figure 13 for *exo*-himic anhydride.

Figure 13 Annotated ^13^C NMR spectrum of exo-himic anhydride, conducted in CDCl_3_ at 298 K.

Figure 14 Annotated HSQC NMR spectrum for exo-himic anhydride, conducted in CDCl_3_ at 298 K.

Having already assigned the ^1^H NMR spectrum for *exo*-himic anhydride, the assignment of the ^13^C NMR is simple using the HSQC spectrum (Figure 14) which correlates the ^1^H and ^13^C signals. The signals on the HSQC spectrum are labelled as ‘δ ^1^H signal, δ ^13^C signal’ as some of the ^13^C peaks are very close together and difficult to tell apart.

# Equipment

NMR data were collected on a Bruker AV II MHz spectrometer, at room temperature, and spectra were referenced to the residual solvent signal of the NMR solvent used. Spectra were processed using ACDLABS software. ^1^H NMR data presented in format: ^1^H NMR (Frequency, Solvent): δ = shift in ppm (coupling pattern, *J* = coupling constant, number of protons, structure assignment). ^13^C NMR data presented in format: ^13^C NMR (Frequency, Solvent): δ = shift in ppm (structure assignment).

Single crystal XRD data was collected on a Rigaku Oxford Diffraction SuperNova A S2 single crystal diffractometer using a Cu radiation source.

IR spectra were obtained on an attenuated total reflectance IR-Affinity-1S Shimadzu spectrometer and measurements were performed on neat samples. Data from IR spectra are presented in format: IR: v_max_/cm^-1^ peak (functional group assignment).

GC-MS data was collected on an Agilent 6890 series GC with MS detector. *Exo*-isomer percentage was calculated from the integration of the area under each peak as determined by the software. Mass spectra data reported in format: m/z: molecular ion.

# Student worksheet

Complete the following table; write down your deductions and identify the protons

| **Chemical Shift (ppm)** | **Integration** | **Multiplicity of Peak** | **Coupling Constant (Hz)** | **Deductions** | **Identity of Peak** |
| --- | --- | --- | --- | --- | --- |
|  |  |  |  |  |  |
|  |  |  |  |  |  |
|  |  |  |  |  |  |
|  |  |  |  |  |  |
|  |  |  |  |  |  |

# Student worksheet - answer

Complete the following table; write down your deductions and identify the protons

| **Chemical Shift (ppm)** | **Integration** | **Multiplicity of Peak** | **Coupling Constant (Hz)** | **Deductions*^a^*** | **Identity of Peak** |
| --- | --- | --- | --- | --- | --- |
| 1.46 | 1 | dt | 1.0, 12 |  | H-3” |
| 1.70 | 1 | d | 3.0, 12 |  | H-3’ |
| 3.03 | 2 | d | 1.0 |  | H-4 |
| 3.49 | 2 | t | 4 |  | H-2 |
| 6.36 | 2 | t | 4 |  | H-1 |

*a* for deductions see previous section and adapt as required for workshop

# References

(1) Craig, D. The Rearrangement of Endo-3,6-Methylene-1,2,3,6-Tetrahydro-Cis-Phthalic Anhydride. *J. Am. Chem. Soc.* **1951**, *73* (10), 4889–4892. https://doi.org/10.1021/ja01154a124.

(2) Long, T. R.; Maity, P. K.; Samarakoon, T. B.; Hanson, P. R. ROMP-Derived Oligomeric Phosphates for Application in Facile Benzylation. *Org. Lett.* **2010**, *12* (13), 2904–2907. https://doi.org/10.1021/ol1006604.

(3) Biagini, S. C. G.; Gibson, V. C.; Giles, M. R.; Marshall, E. L.; North, M. Copolymerization of Amino Acid and Amino Ester Functionalized Norbornenes via Living Ring-Opening Metathesis Polymerization. *J. Polym. Sci. Part A Polym. Chem.* **2008**, *46* (24), 7985–7995. https://doi.org/10.1002/pola.23098.

(4) Shehata, S.; Serpell, C. J.; Biagini, S. C. G. Architecture-Controlled Release of Ibuprofen from Polymeric Nanoparticles. *Mater. Today Commun.* **2020**, *25*, 101562. https://doi.org/10.1016/j.mtcomm.2020.101562.

(5) Birchall, L. T.; Shehata, S.; McCarthy, S.; Shepherd, H. J.; Clark, E. R.; Serpell, C. J.; Biagini, S. C. G. Supramolecular Behaviour and Fluorescence of Rhodamine-Functionalised ROMP Polymers. *Polym. Chem.* **2020**, *11* (32), 5279–5285. https://doi.org/10.1039/d0py00799d.

(6) Matson, J. B.; Grubbs, R. H. Synthesis of Fluorine-18 Functionalized Nanoparticles for Use as in Vivo Molecular Imaging Agents. *J. Am. Chem. Soc.* **2008**, *130* (21), 6731–6733. https://doi.org/10.1021/ja802010d.

(7) Karplus, M.; Anderson, D. H. Valence-Bond Interpretation of Electron-Coupled Nuclear Spin Interactions; Application to Methane. *J. Chem. Phys.* **1959**, *30* (1), 6–10. https://doi.org/10.1063/1.1729943.

(8) Cambridge Crystollagraphic Data Centre executableshttps://www.ccdc.cam.ac.uk/support-and-resources/downloads/.

(9) Gianopoulos, C. G.; Zarychta, B.; Cenedese, S.; Zhurov, V. V.; Pinkerton, A. A. Experimental and Theoretical Electron Density Determination for Two Norbornene Derivatives: Topological Analysis Provides Insights on Reactivity. *J. Phys. Chem. A* **2016**, *120* (23), 4059–4070. https://doi.org/10.1021/acs.jpca.6b03787.

(10) Haasnoot, C. A. G.; de Leeuw, F. A. A. M.; Altona, C. The Relationship between Proton-Proton NMR Coupling Constants and Substituent Electronegativities-I. An Empirical Generalization of the Karplus Equation. *Tetrahedron* **1980**, *36* (19), 2783–2792. https://doi.org/10.1016/0040-4020(80)80155-4.
